# Origins of the seed: The “golden-trio hypothesis”

**DOI:** 10.3389/fpls.2022.965000

**Published:** 2022-08-29

**Authors:** Shu-Nong Bai, Guang-Yuan Rao, Ji Yang

**Affiliations:** ^1^College of Life Sciences, Peking University, Beijing, China; ^2^School of Life Sciences, Fudan University, Shanghai, China

**Keywords:** seeds, origin, seed program, pteridophytes, “golden-trio hypothesis”

## Abstract

The seed is an evolutionary innovation in the plant kingdom. While human civilization depends heavily on seed production, how the seed trait emerged remains elusive. In this opinion article, a “golden-trio hypothesis” is proposed based on our investigations of *LEC1* gene functions in *Adiantum capillus-veneris*. This hypothesis posits that a “seed program” arose from spatiotemporal integration of three key components: assimilate flow, ABA-mediated stress responses, and stress-induced *LEC1* expression. Thus, the evolutionary innovation of seeds should be considered not a simple event resulting from new genes; rather, it represents the outcome of a series of physiological and morphological innovations that emerged prior to and regardless of the origin of the seed program. This new perspective could help us tackle some long-standing questions around the puzzling origin of seeds.

## Introduction

Twenty years ago, one afternoon in early summer, two of the authors of this article discussed evolutionary developmental biology (“evo-devo”) in the corridor linking the main building housing the College of Life Sciences and its annex. One of them was vacationing in Beijing before an upcoming journey to the United Kingdom as a visiting scholar, following a previous collaboration with his colleagues at Australia National University. During this time, evo-devo was starting to become popular in plant biology. In this conversation, they reasoned that committing to such research meant focusing on traits that represent key innovations, such as vascular tissue, seeds, and flowers, in the plant kingdom. Among such key innovations, they preferred to study the origins of seeds. The rationale was simple, that in Arabidopsis, knocking out *LEAFY COTYLEDON1* (*LEC1*) resulted in skipping a “seed program” characterized by assimilate accumulation, desiccation, and dormancy along with the embryo development (Meinke et al., 1994; West et al., 1994; Lotan et al., 1998; Harada, 2001). The *lec1* mutant phenotype somehow resembles what is observed in ferns, in which early embryo development continues from zygote division to seedling growth. While one of the authors talked about it for fun, the other took the idea more seriously. Before his departure to the United Kingdom, he asked a graduate student in his lab to perform a sequence comparison among *LEC1*-related genes from green algae to land plants. This was how the story detailed here started.

## Seeds, seed development, and seed origin

A seed is a morphologically complicated structure. It consists of an embryo (together with a distinguishable endosperm in some angiosperms), which is a multicellular structure derived from the newly formed zygote, and a seed coat, a sporophytic tissue derived from the integuments of a maternal ovule. The seed is a hallmark structure that distinguishes spermaphytes (including gymnosperms and angiosperms) from pteridophytes (including ferns and lycophytes) and bryophytes.

**Figure 1:**
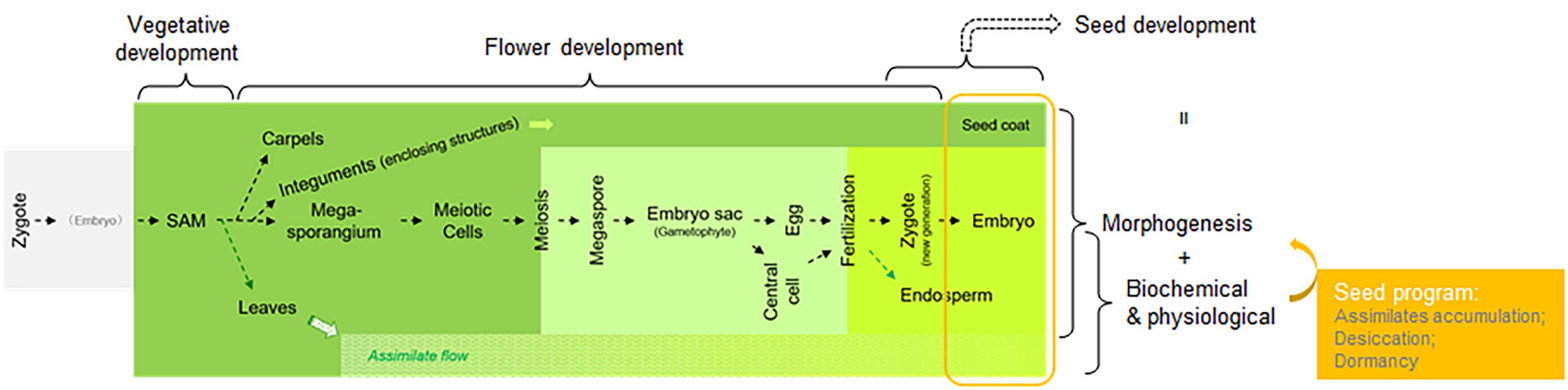
A diagram illustrating the meaning of the “seed program” in the context of the angiosperm life cycle and mainstream study of seed development. Different stages of the angiosperm life cycle are assigned as different research topics, such as vegetative, flower, and seed development, with some overlaps. The sporophyte of the plant, derived from a zygote, is labeled in dark green; the gametophyte is pale green; and the sporophyte of the new generation enclosed within the maternal sporophyte is bright green. The assimilate flow is labeled.

Since seeds are a major food resource, the control of seed size and assimilate accumulation have been intensively investigated, as have post-harvest methods for seed storage that maintain freshness and prevent sprouting. The studies on seed development, mostly with angiosperms as the experimental system, can be roughly categorized into two groups: morphological characterization and physiological analysis. Before the 1980s, physiological studies on seed development mainly focused on the analysis of nutritional composition, assimilate accumulation during embryogenesis, and conditions affecting seed dormancy, among others (Harada, 1997). Once forward genetics gained popularity, especially using Arabidopsis as a model, many genes involved in seed development were identified through mutant screens (Koornneef et al., 1984; Koornneef and Meinke, 2010). Given that a “seed” is the final outcome of developmental processes for sporophytic tissues (e.g., megasporangium and integuments of the ovule in Arabidopsis), gametophytic tissues (e.g., megaspore and gametophyte, including embryo sac differentiations, which are enclosed in the megasporangium), and the new generation of sporophytic tissues (*via* fertilization and embryogenesis), genes identified as being involved in those processes specifically have not been considered “seed genes.” Thus, only genes affecting differentiation steps occurring after the formation of seed-related structures, e.g., integuments and embryos, have been considered seed genes. Among these identified seed genes, in addition to those affecting embryogenesis, endosperm differentiation, and seed coat differentiation, there was a group affecting assimilate accumulation, desiccation, and dormancy, i.e., the biochemical and/or physiological processes that accompany embryo and seed coat morphogenesis. Collectively, these three processes (assimilate accumulation, desiccation, and dormancy) compose seed maturation or what has been called the “seed program” (Wobus and Weber, 1999; Figure 1). *LEC1* plays an important role in the processes underlying the seed program (Harada, 2001).

According to phylogenetic analysis of photoautotrophic organisms, the seed is a relatively new trait that emerged around 200 million years ago (Herr, 1995; Taylor et al., 2009), at a time when non-seed plants such as bryophytes and pteridophytes had already occupied land for over 300 million years. Seed origin is therefore a long-standing question to plant biologists: When, from where, and how did the seed trait emerge?

**Figure 2:**
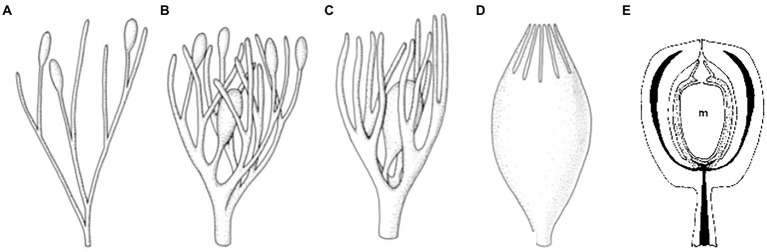
A diagram of two events closely related to the “retention” theory regarding seed origin. **(A–D)** Putative process of integument origin during evolution according to the “telome theory.” **(A)** Oval-shaped megasporangia are located at the tips of the telomes/branches. **(B,C)** Several telomes/branches were coopted as surrounding tissues to enclose the dominant/fertile megasporangium. **(D)** The surrounding tissues merged into a structure designated as the integument (often consisting of two layers in angiosperms; modified from Taylor et al., 2009). **(E)** Illustration of a stalked ovule of *Callospermarion*. “m” refers to a megaspore in the megasporangium (indicated by the dotted region) enclosed by the integument with vascular bundles represented by the black strips (modified from Herr, 1995).

There are diversified hypotheses and terminologies around the origin of seeds—traditionally referred to as the origin of the “seed habit”—based on morphological comparisons and fossil investigations. According to the widely accepted “retention theory” (Herr, 1995; Taylor et al., 2009; Figure 2), a seed is derived from the retention of a megasporangium that differentiated at the tip of a “telome” (a general term describing the diversified shape of branches). Externally, the retained megasporangium is enclosed by surrounding structures that emerged from the telome prior to the megasporangium (Figures 2A–D). These surrounding structures are designated as integuments of the ovule at its early developmental stages and later as the seed coat. Internally, the retained megasporangium can continuously receive nutrition from the rest of the sporophyte (Figure 2E).

In contrast to non-seed plants, e.g., pteridophytes and bryophytes, differentiation of the non-photoautotrophic gametophytes (the haploid multicellular structures derived from spores) in seed plants was physically compressed within the sporangia, for both mega- and micro-sporangia. Within the megasporangium, the egg formed, was fertilized, and then differentiated from a zygote to an embryo. The embryo received its nutritional supply from the sporophytes where the telomes/branches differentiated, rather than from the gametophytes as in bryophytes and pteridophytes.

While it remains elusive how the different parts, such as megasporangium and embryo were integrated into one unit recognized as a seed, it seems that all these parts have counterparts in non-seed plants, based on comparative morphological and paleobotanical studies. However, no clues can be found from such studies on what caused megasporangium retention at the tip of the telome/branch in the first place, how the haploid gametophyte compressed within the megasporangium acquired its nutritional supply from the diploid sporophyte, and under what conditions the enclosed megasporangium together with the embryo detached or abscised from the telome/branch. From this perspective, it is clear that to solve the mystery of seed origin, one must explore new investigation strategies, in addition to those developed in traditional comparative morphology and paleobotany.

## The *lec1* mutant phenotype and a naive hypothesis on seed origin

**Figure 3:**
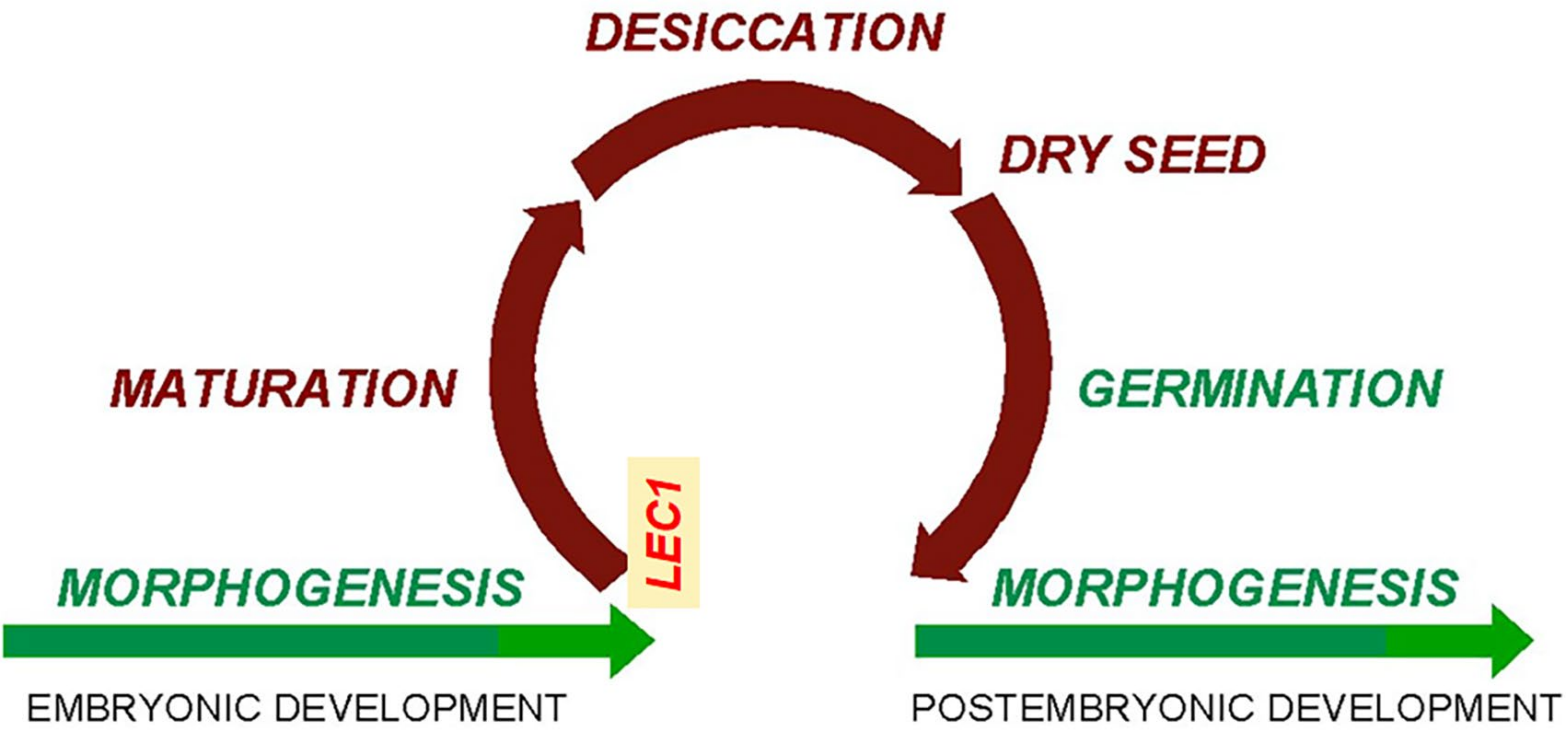
Cycle of seed maturation and germination in higher plants. Relationship between morphogenesis/embryonic development and seed maturation, suggesting that seed maturation interrupts the morphogenesis process (figure courtesy of J. Harada; modified with the addition of “LEC1” to the left turning point).

The *lec1* mutant was first reported in 1992 as a homeotic mutant with cotyledons resembling true leaves (Meinke, 1992). However, the function of the *LEC1* gene was soon interpreted as an “embryonic fate” determinant because its overexpression induced somatic embryogenesis rather than seedling formation during tissue culture (Lotan et al., 1998). Alternatively, John Harada has proposed a more sophisticated explanation for the *lec1* mutant phenotype (personal communication): If indeed there is a seed maturation program (henceforth referred to as the “seed program”) that encompasses assimilate accumulation, desiccation, and dormancy, there must be a regulatory mechanism to initiate the program and coordinate the associated complex biochemical and physiological processes. Since all processes in the seed program were abnormal in the *lec1* mutant, the simplest explanation is that the *LEC1* gene plays a key role in initiating and coordinating the seed program (Figure 3). Due to loss of *LEC1* function, the seed program cannot be properly turned on and coordinated, resulting in defective assimilate accumulation, desiccation, and dormancy, although embryo morphogenesis remains essentially normal, i.e., there is a functional embryotic shoot and root (Meinke et al., 1994; West et al., 1994). Derepression of the seed program during morphogenesis in *lec1* allows primordia committed to the cotyledon in the wild type to differentiate into true leaves with trichomes on the epidermis—the characteristic phenotype of the *lec1* mutant that was the inspiration for its name (Meinke, 1992; Lotan et al., 1998).

**Figure 4:**
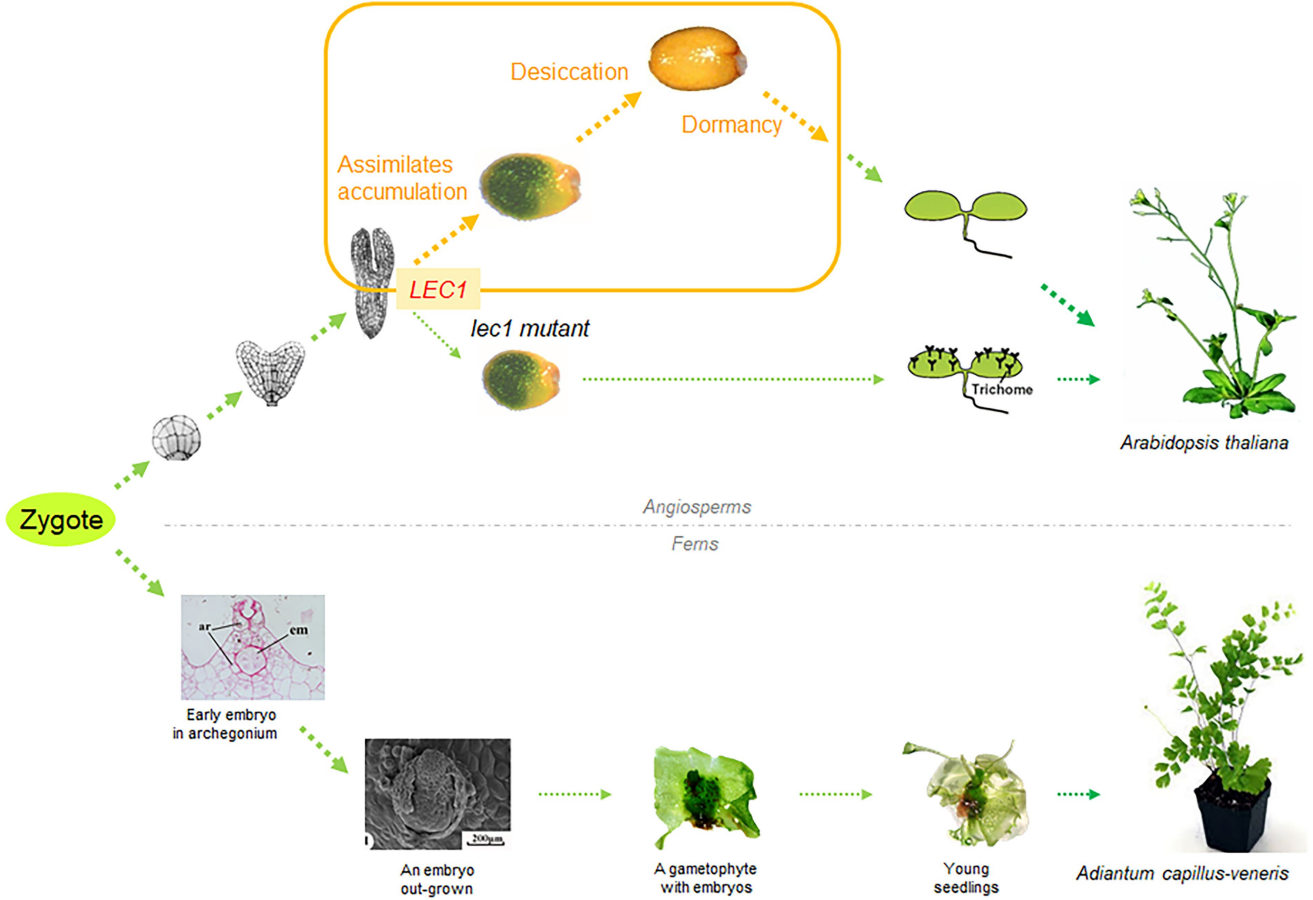
A comparison of early development, from zygote to seedling, between angiosperms and ferns. Angiosperms and ferns are represented by Arabidopsis and *Adiantum capillus-veneris*, respectively. The phenotype of the *lec1* mutant during the embryogenesis resembles that of *Adiantum capillus-veneris* during early development. For a detailed morphological description of *Adiantum capillus-veneris*, see Li et al. (2013).

While many plant geneticists study angiosperms, two of the authors of this article work in the field of plant evolution. They are quite familiar with the life cycles of both seed and non-seed plants and of their differences. Taking this training background into account, it is understandable that while discussing the *lec1* phenotype, they compared the embryogenetic processes of the *lec1* mutant to that of ferns, in which morphogenesis continues without desiccation and dormancy (Figure 4). There are substantial differences in embryogenesis in angiosperms and pteridophytes: In the former, the embryogenesis occurs in the embryo sac embedded in the sporophytic ovules; and in the latter, the embryogenesis occurs in the archegonia of photoautotrophic gametophytes independent of sporophytes. However, it was exciting to hypothesize that the seed program originated due to the *LEC1* gene, if *LEC1* is indeed the key regulator of the seed program. Was this hypothesis experimentally testable? As mentioned above, after their chat in the corridor 20 years ago, one of the authors requested a sequence comparison of *LEC1*-related genes from green algae to land plants.

## *LEC1*-like sequences emerged from pteridophytes and function in *Selaginella* and *Adiantum*

**Figure 5:**
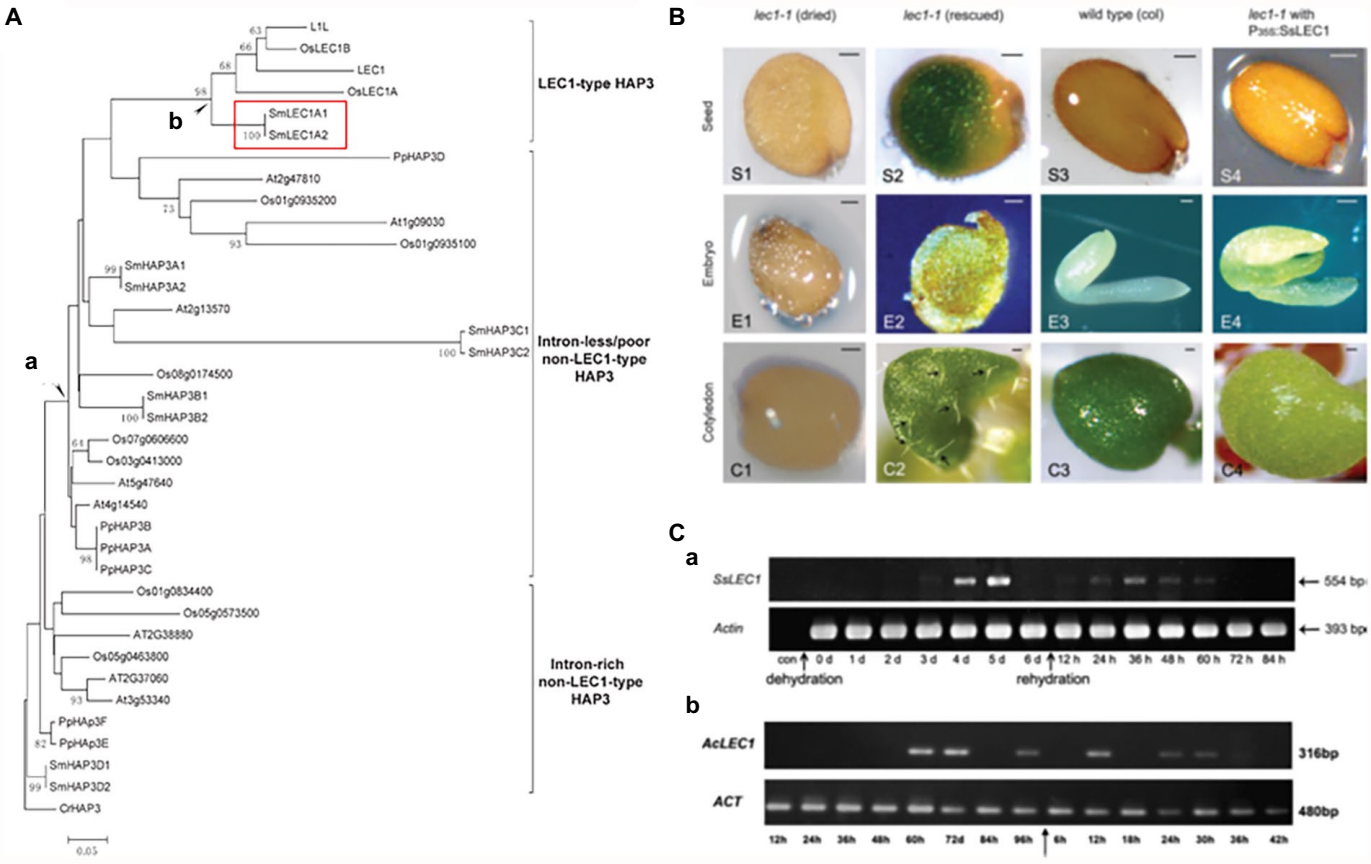
*LEC1*-like gene sequence identification, functional complementation, and stress-inducible expression in lycophytes. **(A)** Phylogeny of HAP3 proteins from different plants, in which the LEC1-type HAP3 (LEC1-like) proteins were identified in the branch labeled “b” (the protein sequences with the prefix “Sm” are from *Selaginella moellendorffii* and are highlighted by a red rectangle). **(B)** *SsLEC1* (LEC1-like from *Seleginella sinensis*) can complement the *lec1* mutant phenotype in Arabidopsis. **(C)** Drought-induced expression of the *LEC1*-like genes in *Selaginella sinensis* (a) and *Adiantum capillus-veneris* (b; modified from Xie et al., 2008).

The genome sequence comparison revealed that *LEC1* belongs to the HAP3 superfamily, together with its closely related non-*LEC1* linage (Yang et al., 2005; Figure 5A). While no *LEC1*-like sequences were found the genomes of algae and bryophytes, *LEC1*-like sequences were present in the lycophyte *Selaginella* (highlighted in Figure 5A), as well as in other lycophyte and fern genomes (Xie et al., 2008). Gene expression and genetic complementation analyses excluded the possibility that the *LEC1*-like sequences identified in the pteridophytes are pseudogenes (Figure 5B) and demonstrated that the *LEC1*-like sequences are expressed under stress conditions (Figure 5C; Fang et al., 2017; Li et al., 2017).

These experimental data led to a perplexing situation: On the one hand, *LEC1*-like genes were relatively new in the context of land plant phylogeny, as they did not exist before pteridophytes. On the other hand, the original function of *LEC1*-like genes was unlikely related to a seed program, as the seed trait is absent in *Selaginella* and *Adiantum*. However, the *LEC1*-like genes are expressed during morphogenetic processes of *Selaginella* and *Adiantum* and functionally complement Arabidopsis *LEC1*. If the function of *LEC1* was interpreted as a key regulator in the seed program, as most previous studies have claimed, such an interpretation obviously is not applicable to that of its similar sequences found in the pteridophytes. If the function of the *LEC1*-like gene in pteridophytes is regarded as ancestral, it becomes necessary to explain how the gene was coopted into the seed program, leading to the emergence of the spermatophytes and functioning as a key regulator of the seed program.

While the experimental data disproved a causal relationship between the origin of *LEC1* and the origin of the seed program, they generated a new opportunity to solve the seed origin mystery from the perspective of the ancestral function of stress-responsive *LEC1*-like genes in pteridophytes.

## *LEC1*-like genes in pteridophytes and the rationales of the “golden-trio hypothesis”

Two important facts about the *LEC1*-like genes were discovered (Xie et al., 2008): First, the *LEC1*-like genes emerged in the pteridophytes, prior to the spermatophytes; and second, the *LEC1*-like genes are induced by stress during pteridophyte morphogenesis. These new facts, together with the known fact that *LEC1* is a key regulator of the seed program in Arabidopsis, support the hypothesis that the function of *LEC1* as a seed program regulator was derived from the ancestral function of stress-responsive *LEC1*-like genes in pteridophytes. Now, another question arises: Why did not the expression of *LEC1*-like genes in the pteridophytes trigger a seed program, even though these sequences were capable of restoring the seed program in the Arabidopsis *lec1* mutant?

There are two possible answers to the above question. First, it is possible that the *LEC1*-like-regulated genes required to organize the seed program regulatory network were absent in the pteridophytes. Second, it is possible that the proper conditions required to induce *LEC1*-like gene expression were not met during gametophyte development in pteridophytes despite the presence of the *LEC1*-like-regulated genes in the genome, and therefore no seed program could be activated.

The first possibility could be easily tested by genome comparison if the genome sequences of pteridophytes were available or through experimental analysis. Current information suggests that a lack of downstream genes is less likely the reason for no seed program in pteridophytes (Han et al., 2017; Li et al., 2017). As for the second possibility, while stress-induced expression of *LEC1*-like genes was found during sporophytic development in both *Selaginella* and *Adiantum*, none was detected during gametophytic development (Fang et al., 2017). As gametophytic development is highly sensitive to dehydration and ABA application, it is necessary to find other conditions to test whether *LEC1*-like genes can be expressed during gametophytic development.

In addition to dehydration and ABA application, hexose sugars were reported to induce *LEC1* gene expression during seed development (Tsukagoshi et al., 2007). This finding not only provides a new agent to be examined for its effect on *LEC1*-like gene expression in pteridophyte gametophytic development, but more significantly, hints to a possible scenario for how the *LEC1* gene was coopted as a key regulator of a future seed program, which will be further elaborated below.

As mentioned previously, seeds are the key structures distinguishing spermatophytes from the pteridophytes and bryophytes. While all land plants possess two multicellular structures, diploid sporophytes and haploid gametophytes (Smith et al., 2010; Bai, 2015), the gametophytes of spermatophytes are compressed in megasporangia, which are “retained” at the tip of the telomes/branches where the megasporangia differentiate. It remains elusive how the megasporangia were retained at the tip of the telomes/branches and why the megaspores were not released, after which gametophytic development and embryogenesis continue within the retained megasporangia. However, two facts were unambiguously observed. First, the retained megasporangia were all located at the extremity of the vascular system. This fact suggests that the retained megasporangia enclosed by the integuments are a sink for assimilate flow (Herr, 1995; Taiz et al., 2014). The second fact is that before the floral structure emerged, megasporangia were mostly aerial. Even though megasporangia were also enclosed by integuments in gymnosperms, the combined entities, called ovules, were also aerial (Taylor et al., 2009; Bai, 2017, 2019). This fact suggests that a mechanism to cope with dehydration would have likely evolved during the differentiation of megasporangia.

**Figure 6:**
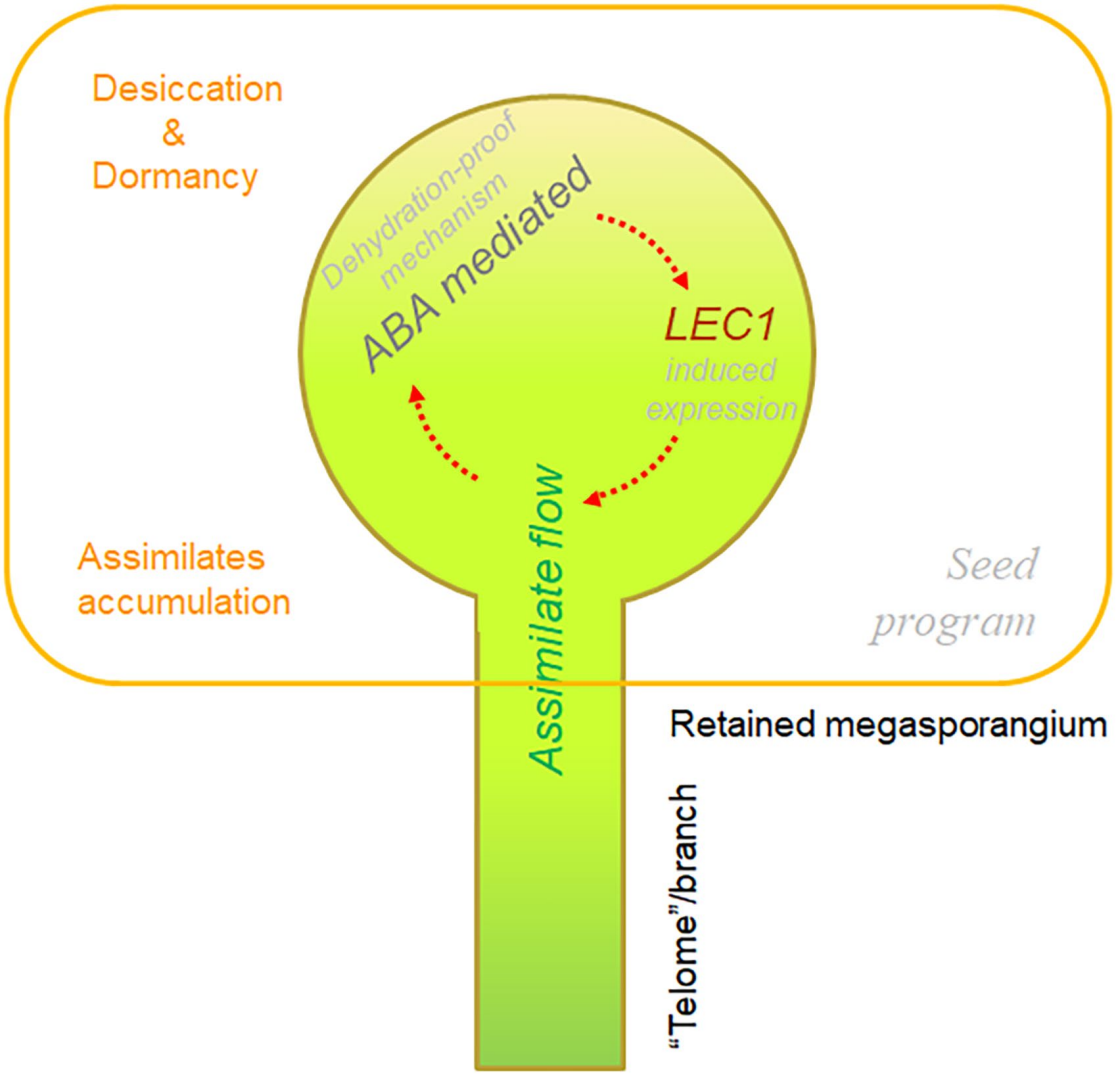
Diagram of the “golden-trio hypothesis” on the origin of the “seed program.” The “golden trio” refers to three indispensable components for seed program: assimilate flow, ABA-mediated dehydration-proof mechanism, and inductive expression of *LEC1*-like genes. Interactions among the three components, together with other factors, triggered the seed program overlapping with embryonic development, resulting in emergence of the seed trait.

Taking the above two facts into consideration, a possible scenario emerges: Regardless of how the megasporangia were retained at the tip of telomes/branches and why the megaspores were not released, gametophytic development and embryogenesis continued inside of the megasporangia, which became a sink for assimilate flow originating from the sporophytes. If the megasporangia were indeed a sink, together with the dehydration-proof mechanism (probably mediated with ABA as revealed from numerous studies in angiosperms, Finkelstein and Gibson, 2002; Weber et al., 2005), it is highly possible that *LEC1*-like gene expression in pteridophytes could be induced by assimilates containing hexoses as well as by the dehydration-proof mechanism (likely mediated by ABA). If the molecular mechanism of *LEC1*-mediated seed program regulation is common amongst Arabidopsis and other angiosperms, it is difficult to reject the possibility that the induced expression of *LEC1*-like genes could trigger a similar seed program during the differentiation of megasporangia in pteridophytes or specifically some ancestors of spermatophytes. If the integration of these three components (i.e., assimilate flow, an ABA-mediated dehydration-proof mechanism, and inducible expression of *LEC1*-like genes) indeed occasionally occurred within the retained megasporangia (possibly already been enclosed by the surrounding structures), perhaps a seed program was eventually established during the evolution of land plants. We describe this scenario for the origin and activation of a seed program in land plants as the “golden-trio hypothesis” (Figure 6).

## Preliminary testing of the golden-trio hypothesis

In the scenario described above, assimilate flow and the ABA-mediated dehydration-proof mechanism were likely indispensable for inducing *LEC1*-like gene expression to activate the seed program. Given that gametophytic development in pteridophytes is highly sensitive to ABA, at least one of the components required for the activation of the seed program must be lacking in these plants. In addition, gametophytes that develop in lycophytes such as *Selaginella* are very limited in size and barely self-sustainable (Schulz et al., 2010), and those in ferns such as *Adiantum capillus-veneris* lack conducting tissue, although the prothallus is self-sustainable. Thus, there is little possibility of extra assimilate accumulation during embryogenesis as storage materials. From this perspective, it is easy to explain why there are *LEC1*-like genes but no seed program in pteridophytes: The conditions required to induce the expression of *LEC1*-like genes during gametophytic development were not met.

If the above reasoning stands, one might ask whether artificially applying assimilates during gametophyte development in pteridophytes would induce the expression of the *LEC1*-like genes and activate a seed program. To answer this question, a new experimental system must be established to meet two criteria: First, gametophytic development in this system must be easy to manipulate under laboratory conditions. Second, it must be possible to effectively assess the biochemical and physiological processes involved in the seed program of the cultivated gametophytes.

Fortunately, *Adiantum capillus-veneris* was demonstrated as a suitable species for establishing the experimental system (Li et al., 2013). Not only could its gametophytic development be manipulated under laboratory conditions, but its representative gene expression and metabolite accumulation were also possible to analyze during gametophytic development. Using such an experimental system, Fang et al. (2017) showed that sugar treatments can induce *Adiantum capillus-veneris LEAFY COTYLEDON1* (*AcLEC1*) expression and trigger the accumulation of storage products during prothallus (gametophyte) development. While it remains to be determined whether the sugar treatments directly caused the increase in *AcLEC1* expression and the accumulation of storage products, these findings provided the first supporting evidence for the golden-trio hypothesis.

## Perspectives

While a clear conclusion about seed origin has not yet been reached, studies of pteridophytes have expanded our understanding of the function of *LEC1* and *LEC1*-like genes, particularly their roles in the seed program. These findings also introduced a new perspective on how to approach the questions surrounding seed origin, in addition to traditional comparative morphology and paleobotany.

How could the new findings from the studies on *LEC1*-like genes in pteridophytes shed light on our understanding of seed origin? While studies demonstrated the existence of functional *LEC1*-like genes in pteridophytes, their inducible expression during gametophyte development and the existence of *LEC1*-downstream genes in *Adiantum capillus-veneris* strongly suggested that seed origin is not an evolutionary innovation that was directly switched on by *de novo* gene birth. There were at least two innovations for the seed trait to ultimately emerge: One is the retained megasporangia development, and the other is the interwoven biochemical and physiological processes designated as the seed program. For the latter, the findings from pteridophyte studies suggest that while some genes such as *LEC1*-like are key regulators of the seed program, other pre-existing components are indispensable for the expression of *LEC1*-like genes during gametophyte development. In short, the emergence of the seed trait as a key evolutionary innovation most likely resulted from complicated interactions and the integration of pre-existing components.

A recently published work provides strong support for that hypothesis; based on the genome sequence and transcriptome analysis of *Adiantum capillus-veneris*, Fang et al. (2022) determined that *AcdLEC1* (previously designated as *AcLEC1*) is not only expressed during homosporangium development, but also resides at a hub of a regulatory network that is conserved in the Arabidopsis seed program. The work further elucidated the function of *AcdLEC1* in pteridophytes, which consistent to the previous finding that the *AcdLEC1* expressed in sporophytes under stress conditions (Xie et al., 2008). The similarity of regulatory network between the *Adiantum capillus-veneris* homosporangium development and Arabidopsis seed program provides a new evidence to what was predicted in the golden-trio hypothesis for origin of seed program.

**Figure 7:**
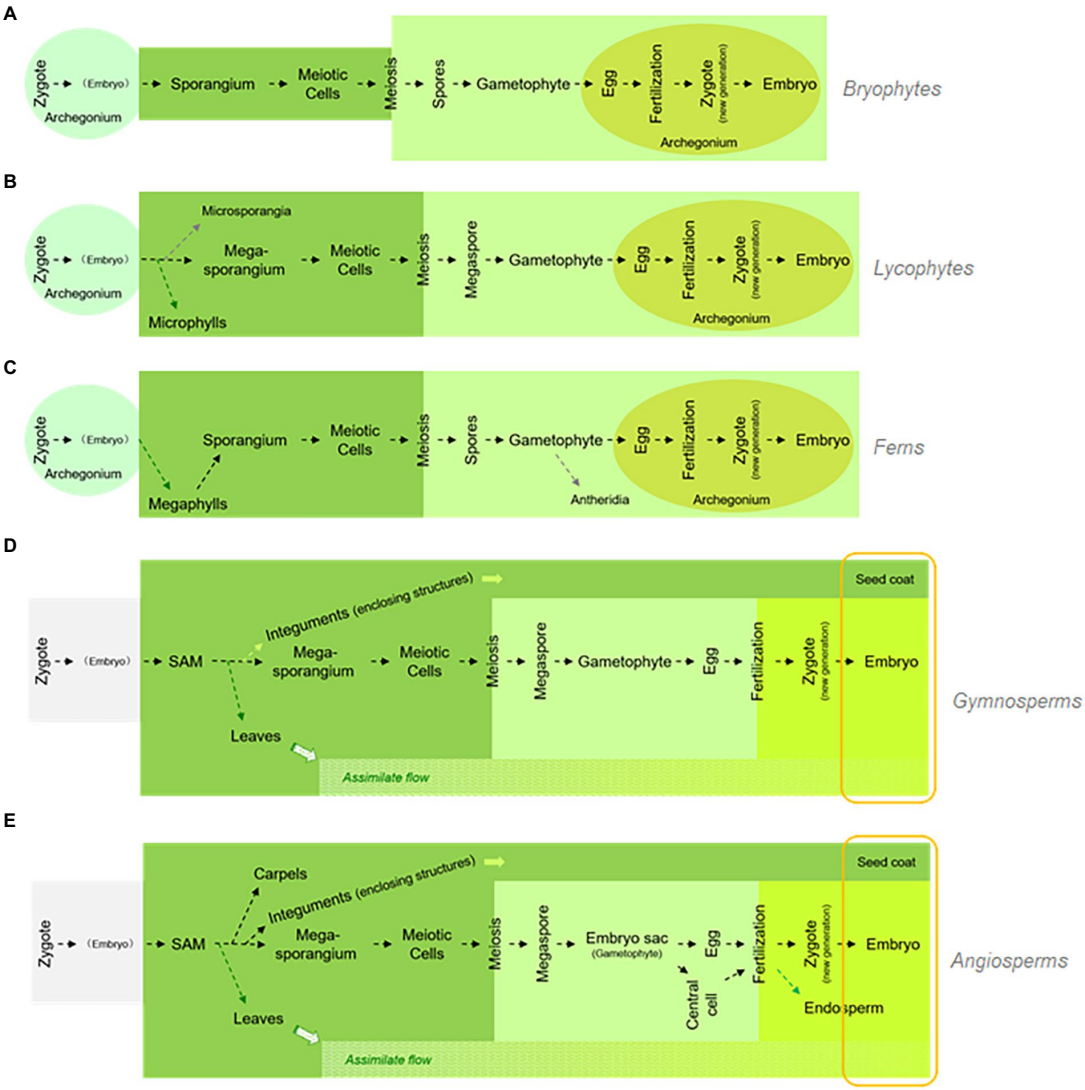
Life cycle comparison among five major land plant groups and key evolutionary innovations. The five major land plant groups are bryophytes, lycophytes, ferns, gymnosperms, and angiosperms, labeled from **A–E**, respectively. While the successful development of a seed plant requires integration of multiple evolutionary innovations, these innovations may emerge independently, regardless of seed origin (labeled in round-cornered rectangular, compares to Figure 1).

Integrating the above-mentioned findings from the study of pteridophytes, along with our knowledge on the function of *LEC1* genes and the comparative morphology/paleobotany of seed origin, we developed the golden-trio hypothesis to explain how stress-inducible *LEC1*-like genes were coopted during gametophytic development to become key regulators of the so-called “seed program.” In this hypothesis, retention of megasporangia development at the tip of the telomes/branches was indispensable for seed program emergence. Under such circumstances, assimilate flow and the ABA-mediated dehydration-proof mechanism were present simultaneously and could induce the expression of the *LEC1*-like genes in developing megasporangia as well as in gametophytes/embryos compressed within the retained megasporangia. Figure 7 compares the life cycle of bryophytes (a), pteridophytes (including lycophytes and ferns; b, c), and spermatophytes (including gymnosperms and angiosperms; d, e). This diagram illustrates the emergence of key components related to seed origin with respect to the phylogeny of land plants.

Traditionally, many evolutionary innovations are included under the topic of “seed origin,” or “origin of seed habit,” such as heterospory, retention of megasporangium, origin of integuments, and even pollen. However, based on the findings from studies on *LEC1*-like gene function, it seems that the innovations recognized as indispensable for seed origin might not have emerged exclusively for the origin of the seed trait or “seed habit.” Rather, these innovations originally emerged regardless of seed origin and then interacted by chance with other innovations. Among those chance interactions remained a few with adaptive advantages, which were designated as “seed plants.” Wang and Bai (2019) have proposed a possible scenario regarding the transition from homospory to heterospory. Similar adaptive advantages of this transition might underlie the retention of the megasporangia in sporophyte development, which ultimately led to the emergence of the spermatophytes. Based on the conservation of LEC1-centered regulatory networks between *Adiantum capillus-veneris* homosporangium development and the Arabidopsis seed program (Fang et al., 2022), such LEC1-centered regulatory networks found in *Adiantum capillus-veneris* might have been available for integration with other evolutionary innovations, resulting in the origin of seeds, which would represent the kind of “tinkering” event suggested by Jacob (1977).

Twenty years have passed since the original idea to explore the role of *LEC1* in seed origin. Although the first hypothesis on the relationship between *LEC1* and seed origin was proven to be oversimplified, it in principle fell within the scope of classic evo-devo. One lesson should be learned from the study of *LEC1*-like genes in pteridophytes: There may be a disconnect between the origin of traits and the genes responsible for these traits. Traditionally, genes and their functions were designated by mutant phenotype analysis in model organisms. When a gene was demonstrated as indispensable to a trait, it was inferred that the trait was derived from the origin of that gene. However, with more genomic information available, an increasing number of examples indicate that genes tend to exist prior to the origin of the traits they regulate. These examples include not only the *LEC1*-like genes, but also genes previously annotated as nerve-specific in sponges that lack a nervous system (Musser et al., 2021). These examples suggest that the original functions of genes might not be what is inferred based on the mutant phenotype in model organisms. Evo-devo researchers should keep in mind that there can be a disconnect between genes and traits pertaining to evolutionary innovation. In conclusion, the findings of the inducible *LEC1*-like gene expression and assimilate accumulation in *Adiantum capillus-veneris* gametophyte development provide a new opportunity to explore the mechanisms of seed origin.

## Author contributions

All authors listed have made a substantial, direct, and intellectual contribution to the work and approved it for publication.

## Funding

The studies on the *LEC1*-like genes were supported by National Natural Science Foundation of China grant no. 30370092 to JY and grant no. 91231105 to G-YR.

## Conflict of interest

The authors declare that the research was conducted in the absence of any commercial or financial relationships that could be construed as a potential conflict of interest.

## Publisher’s note

All claims expressed in this article are solely those of the authors and do not necessarily represent those of their affiliated organizations, or those of the publisher, the editors and the reviewers. Any product that may be evaluated in this article, or claim that may be made by its manufacturer, is not guaranteed or endorsed by the publisher.
